# The utility of nonhuman primate models for understanding acute HIV-1 infection

**DOI:** 10.1097/COH.0000000000000920

**Published:** 2025-03-27

**Authors:** Matthew S. Parsons, Diane L. Bolton

**Affiliations:** a Walter Reed Army Institute of Research - Armed Forces Research Institute of Medical Sciences, Bangkok, Thailand; b U.S. Military HIV Research Program, CIDR, Walter Reed Army Institute of Research, Silver Spring; c Henry M. Jackson Foundation for the Advancement of Military Medicine, Inc., Bethesda, Maryland, USA

**Keywords:** acute HIV-1 infection, chronic HIV-1 infection, nonhuman primates, simian immunodeficiency virus, simian-human immunodeficiency virus

## Abstract

**Purpose of review:**

Nonhuman primate (NHP) models of HIV-1 infection provide complementary experimental pathways for assessing aspects of acute HIV-1 infection (AHI) that cannot be addressed in humans. This article reviews acute infection studies in SIV-infected or SHIV-infected macaque species over the previous 18 months.

**Recent findings:**

Reviewed studies examined the dynamics of replication-competent viral reservoir establishment during early infection, reservoir maintenance throughout therapy, and factors influencing viral rebound after treatment cessation. Also discussed are acute infection events in the central nervous system and liver and potential links between these events and manifestations of comorbidities during chronic infection. Additional studies addressed how occurrences during acute infection impact the development of natural viral control or posttreatment control. Another report evaluated treatment during acute infection with broadly neutralizing antibodies with enhanced ability to engage innate immune cells, highlighting the ability of this early intervention to shape innate and adaptive antiviral responses.

**Summary:**

NHP models of HIV-1 infection are a fundamental research tool for investigating AHI events. These models enable detailed pathogenesis characterization and the testing of hypothesis-driven strategies for altering disease courses through interventions during AHI, including targeting viral persistence and comorbidities that persist throughout chronic infection.

## INTRODUCTION

Acute HIV-1 infection (AHI) best describes the interval between viral exposure and the development of antiviral antibodies. The period is characterized by infection of target cells, high viremia, systemic viral dissemination and replication, immune activation, and rapid depletion of CD4^+^ T cells in the gut-associated lymphoid tissue (GALT) [1–12]. AHI is a vital area of research, as events occurring during this period inform efforts to prevent and treat HIV-1 and understand HIV-1 pathogenesis. In particular, interventions during AHI or among people who initiated antiretroviral therapy (ART) during AHI represent promising opportunities for HIV-1 therapeutics.

Two key processes initiated during AHI have long-term consequences for the host: the establishment of the HIV-1 reservoir and immunologic perturbations. A reservoir of latent replication-competent virus is seeded within immune cells during AHI, persists throughout ART and gives rise to rebound viremia upon treatment interruption, necessitating lifelong ART in most people [13–17]. Detailed understanding of latent reservoir dynamics and characteristics informs the development of HIV-1 cure strategies. In addition, accumulating evidence implicates the events of AHI in health complications that endure in ART-treated people living with HIV-1 (PLWH) during chronic HIV-1 infection. For example, it is hypothesized that the rapid depletion of GALT CD4^+^ T cells during AHI and their incomplete restoration during ART initiated in chronic infection contributes to the deterioration of gut tissue integrity, microbial translocation, and chronic immune activation that persists in ART-treated PLWH [18]. Precisely defining how the events of AHI contribute to residual health complications in ART-treated PLWH will facilitate the development of effective therapeutic interventions.

Given the significance of AHI, identifying people at risk of acquiring HIV-1 or who have recently acquired HIV-1 is a major focus of clinical research efforts [6,19–21]. Although these efforts have been successful, studying AHI in humans is inherently challenging. First, many PLWH are diagnosed during chronic infection, and it is difficult to identify people during AHI. Second, invasive procedures to study relevant anatomical sites are impractical or impossible. Finally, interventions in PLWH can be protracted due to the need to prepare reagents under good manufacturing practices (GMP) and to obtain approval from the US Food and Drug Administration (FDA). 

**Box 1:**
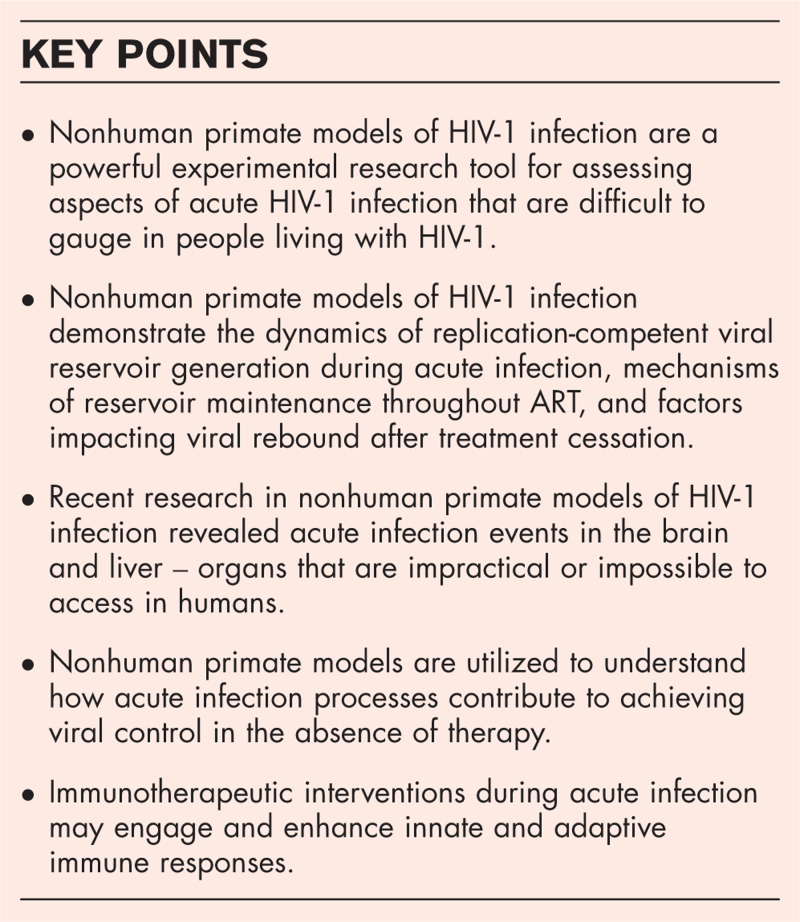
no caption available

**FIGURE 1:**
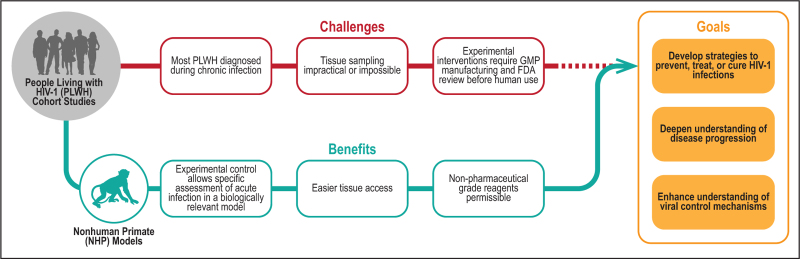
Nonhuman primate (NHP) models of HIV-1 infection provide a means of studying acute HIV-1 infection (AHI) to overcome the challenges associated with assessing this stage of HIV-1 infection in people living with HIV-1 (PLWH). The relative rapidity and ease of studying acute infection in NHP models complements research in cohorts of PLWH and enhances our understanding of the manifestations of natural infection and mechanisms of viral control. Together, research in PLWH and NHP models of HIV-1 infection drives the development of novel strategies to prevent, treat, and cure HIV-1.

Many of these challenges can be overcome through the utilization of animal models of HIV-1 infection. Nonhuman primate (NHP) models in particular provide a parallel experimental pathway for exploring the relationship between the events of AHI and subsequent virologic and immunologic processes (Fig. 1). Multiple NHP species can be infected with simian immunodeficiency viruses (SIV) and chimeric simian-human immunodeficiency viruses (SHIV) that mimic many aspects of AHI, disease progression, and successful treatment with ART [22,23,24^▪▪^,^25^,^26^,^27^^▪▪^,^28^–^31^]. The relative merits of different NHP species and virus strains commonly used in preclinical HIV-1 vaccine and immunoprophylaxis studies is reviewed comprehensively elsewhere [23]. For HIV-1 therapeutic intervention and reservoir/pathogenesis studies, macaque infections with SHIV or SIV strains that replicate robustly in the host without evidence of spontaneous control are generally considered the gold standard. The most common species used in HIV-1 research is Indian-origin rhesus macaques. Unless otherwise stated, this is the model system used in the studies highlighted below. SHIVs, which incorporate the HIV-1 *env* gene inserted into an SIV backbone genome, enable evaluation of interventions specific to HIV-1 Env, such as vaccines and HIV-1 broadly neutralizing monoclonal antibodies (bNAbs). Although advances in SHIV development and engineering have resulted in SHIVs with improved *in-vivo* replication capacity [29,30], SIV strains have historically achieved more consistent acute viral loads and rebound posttreatment interruption, sustained elevated chronic viral loads, and progressive pathogenicity, more closely resembling that of HIV-1 infection in humans. Consideration of these factors is important in the design and interpretation of NHP studies, particularly in relation to HIV-1 pathogenesis and interventions aimed at HIV-1 therapy and cure.

To gauge recent advances in the study of AHI using NHP models of HIV-1 infection, we performed a PubMed search for articles published in the preceding 18 months (i.e. 1 June 2023 to 30 November 2024). This manuscript discusses a curated selection of articles from this period. We focused on advances in understanding viral reservoir generation during acute infection and maintenance following early ART initiation, the impact of early infection events on viral rebound following treatment interruption, acute infection events in the central nervous system (CNS) and liver, the relationship between acute infection processes and spontaneous viral control, and the effect of bNAb interventions administered during acute infection.

## STUDYING VIRAL RESERVOIR FORMATION, MAINTENANCE, AND REBOUND IN NONHUMAN PRIMATE MODELS

A significant barrier to achieving an HIV-1 cure is the persistence of a replication-competent viral reservoir throughout ART that almost invariably rebounds following treatment cessation. Studies in both PLWH and NHPs demonstrate that this reservoir is established early during acute infection [14,17,26]. However, many questions remain about the dynamics of viral reservoir establishment, the relative roles of cellular proliferation and ongoing viral replication during ART for reservoir maintenance, and factors impacting viral rebound following treatment interruption.

To address questions about the dynamics of reservoir establishment, Keele *et al.*[24^▪▪^] studied macaques infected with barcoded SIVmac239M, followed by ART initiation on days 3, 4, 5, 6, 7, 9, or 12. All animals were viremic before ART, and the study design permitted assessment of the impact of treatment commencement at increasing viral loads. The sequence-tagged synthetic SIV stock enabled distinct variants within an otherwise isogenic swarm to be tracked and quantified longitudinally. After 1 year of treatment, ART was interrupted, and rebound viremia was monitored. Animals that received ART the earliest (i.e. 3–4 days postinfection) exhibited the lowest probability of rebound (1/8), the longest time to viral rebound (50 days posttreatment interruption), and rebound viremia derived from a single lineage. Conversely, treatment initiation 7–12 days postinfection resulted in rebound 7–16 days post-ART cessation in all animals and with 3–28 rebound lineages. ART initiation in the middle window (5–6 days postinfection) resulted in an intermediate phenotype: 100% rebound within 14–50 days comprised of one to two lineages. Of note, these results differ from those previously published by Whitney *et al.*[28], in which uniform viral rebound occurred in macaques initiating ART 3 days postinfection. Differences in the design of these studies might explain the discordant observations. Challenge via the intrarectal route in the Whitney *et al.* study versus intravenous in Keele *et al.* may contribute to distinct reservoir establishment dynamics. Also, the shorter treatment duration in the Whitney *et al.* study may have been insufficient for infected cell decay. These data highlight the highly dynamic nature of acute infection with respect to persistent reservoir seeding probability and size.

To confirm that the seven nonrebounding animals that received ART 3–4 days postinfection did not harbor a replication-competent viral reservoir, the authors performed a series of repeated CD8α depletions [24^▪▪^]. This strategy removes potential antiviral effector cells in the host and drives CD4^+^ T-cell proliferation. Despite this intervention, no rebound was noted, suggesting that early infected cells are highly labile, and reservoir formation during this period is stochastic. Cells infected before the initiation of ART 3-4 days postinfection may be less likely to establish a latent infection, clonally expand, or survive the 1-year ART treatment period. Keele *et al.* proposed a three-phase model of replication-competent viral reservoir accumulation during acute infection. First, there is an early stochastic phase 3–4 days postexposure; initiating prolonged treatment during this window largely prevents viral rebound after treatment cessation. Second, there is an exponential phase between 5 and 7 days postinfection, during which the replication-competent viral reservoir rapidly accumulates. Finally, a saturation phase occurs between days 9 and 12, where the growth of the replication-competent viral reservoir slows.

Another area of active research pertains to the possibility of ongoing viral replication during ART contributing to reservoir maintenance. Immonen *et al.*[27^▪▪^] added to the body of evidence that viral replication does not occur under suppressive ART, including in lymphoid tissues. Specifically, macaques infected with barcoded SIVmac239M followed by ART initiation early after infection (i.e. 10–27 days) exhibited similar viral sequences in peripheral blood mononuclear cells and lymphoid tissues longitudinally during ART, with no evidence of viral evolution from ongoing viral replication during treatment periods of up to 3 years. Viral replication in tissues not sampled was deemed unlikely through sequence analysis of rebound viremia following treatment interruption, which also exhibited no evidence of viral evolution. These data support the widely held view that viral replication is unlikely during suppressive ART in most people and that it is not a contributing factor to viral reservoir maintenance [32–35].

Finally, a series of studies aimed to identify factors contributing to the occurrence and magnitude of viral rebound after treatment interruption. Aid *et al.*[36^▪^] determined peripheral biomarkers present before treatment interruption that predict viral rebound in SIVmac251-infected macaques after treatment cessation. These included pro-inflammatory cytokines, macrophage activation, and persistent antigen stimulation during ART. Pinkevych *et al.*[37^▪^] performed mathematical modeling using data from 124 SIV-infected macaques from 10 previous studies conducted at three institutions to assess the impact of the time of ART initiation on posttreatment viral rebound peak and setpoint viremia. Animals initiated ART between 4 and 377 days postinfection. Each day of delay in initiating ART over the first 3 weeks of infection was associated with lower rebound peak and setpoint viremia after treatment interruption. However, after 3 weeks of infection, delays in ART initiation were associated with higher viral rebound after treatment cessation. These findings suggest the existence of a narrow window of opportunity in early infection, during which delayed treatment facilitates the generation of optimal immune responses of potential utility for controlling viral replication following treatment interruption.

These studies provide significant advances in understanding how the replication-competent viral reservoir is generated and maintained, as well as the factors influencing viral rebound after treatment interruption. Potential windows of opportunity for interventions suggest that tailored interventions may benefit individuals who initiate ART at different times relative to recent HIV-1 acquisition. First, initiating prolonged ART in the first 3–4 days of infection may prevent the establishment of a persistent replication-competent viral reservoir. Once the first window of opportunity has passed, delaying ART until 3 weeks postinfection may allow optimal immune response development. As suggested by Pinkevych *et al.*[37^▪^], individuals who initiate treatment during this period might benefit from interventions to hone and expand the immune responses that develop during the pre-ART period. In contrast, individuals who do not start ART until later in infection might benefit from interventions to counteract immune exhaustion and viral escape. Evaluating these concepts in people identified during early acute infection will be informative in developing therapeutic strategies with broader applicability to nonacute infections.

## STUDYING THE CENTRAL NERVOUS SYSTEM DURING ACUTE SIMIAN IMMUNODEFICIENCY VIRUS INFECTION

A significant advantage of NHP models of HIV-1 infection is the ability to sample tissues affected by the virus that are impractical or impossible to access in PLWH. Lymphoid tissues and GALT are major sites of viral replication and are thus frequently included in studies in humans and NHPs [1,9–11,17,24^▪▪^,^25^,^26^,^27^^▪▪^,36^▪^]. Other compartments, however, such as the CNS, remain poorly characterized in PLWH, except for analyses performed on cerebrospinal fluid (CSF) and end-of-life autopsy tissue [1,2,7,8,38].

Neurocognitive impairments are a significant complication for PLWH; even in individuals on suppressive ART, a substantial number experience neurocognitive impairment [39–41]. HIV-1 replication and inflammation are observed within CSF during acute HIV-1 infection [7,8,12]. These early events potentially contribute to establishing the CNS viral reservoir and neurocognitive impairment. Much remains unknown about the phenotypes of lymphocytes and myeloid cells infected and involved in mediating inflammation during acute infection. There is also an unmet need for therapeutics that cross the blood–brain barrier (BBB) and eliminate infected cells. Recent publications assessing CNS CD4^+^ T cells and myeloid cells during acute SIV infection provide valuable insight into the dynamics and infection of these immune cells.

CD4^+^ T cells are present under homeostatic conditions in the mammalian CNS and are hypothesized to contribute to immune surveillance, microglia development, and learning processes [42–44]. Elizaldi *et al.*[45^▪▪^] provided detailed phenotyping of CD4^+^ T cells in the uninfected macaque CNS. The CD4^+^ T-cell landscape in the CSF consists of antigen-experienced CD28^+^CD95^+^ cells. Furthermore, cells with a CCR5^+^CCR7^−^ phenotype, which comprise 4% of antigen-experienced CD4^+^ T cells in blood and lymph nodes, are prevalent within the CSF. The CD4^+^ T cells in the brain commonly express CXCR3, facilitating their migration into the CNS. Like their CSF counterparts, CD4^+^ T cells throughout various CNS regions, including brain parenchyma, choroid plexus stroma, dura mater, and skull bone marrow, frequently express a CCR5^+^CCR7^−^ phenotype, indicating the widespread distribution of putative SIV-susceptible T cells. These cells are similar to tissue-resident memory CD4^+^ T cells observed in the human brain, which exhibit CXCR3 and CCR5 expression [44].

Next, macaques were used to study acute SIVmac251 infection in the CNS. The authors monitored CSF viral loads weekly for 3 weeks in 10 macaques and sacrificed four animals 3 weeks after infection for brain tissue analyses [45^▪▪^]. CSF SIV RNA was detected 1 week after infection and peaked at 2 weeks. CD4^+^ T-cell frequency and CD4 : CD8 T-cell ratio significantly decreased in the CSF 2 and 3 weeks postinfection. Three weeks postinfection, viral RNA and DNA were detected across multiple CNS regions (i.e. white and gray matter from the prefrontal cortex, white and gray matter from the superior temporal sulcus, hippocampus, pituitary, dura mater, skull bone marrow, and choroid plexus stroma). Single-cell RNA sequencing of CD45^+^ cells from the brain showed that viral RNA was primarily found in CD4^+^ T-cell and monocyte clusters. CD4^+^ T-cell depletion was significant in the brain parenchyma, choroid plexus stroma, dura mater, and skull bone marrow 3 weeks after infection compared to uninfected controls. These data highlight that SIVmac251 rapidly accesses and replicates in the CNS and dramatically impacts CD4^+^ T cells. The findings support other studies demonstrating viral RNA in human and NHP CSF CD4^+^ T cells during acute and early infection [7,46], and further implicate CD4^+^ T cells as potential mediators of HIV-1 entry into the CNS [47]. The data also reflect the ability of many SIV (e.g. SIVmac239 or co-infection with SIVdeltaB670 and SIV/17E-Fr) and SHIV (e.g. SHIV-SF162P3, SHIV.C.CH505, and SHIV-1157ipd3N4) strains to access the CNS during acute or early infection [7,48–51].

Further, a cohort of six SIVmac251-infected macaques was subjected to a suboptimal ART treatment plan initiated 3 weeks postinfection and euthanized 42 weeks postinfection to study CNS viral persistence and the impact of suboptimal ART adherence on CD4^+^ T-cell recovery [45^▪▪^]. ART was administered until two successive CSF viral load readings achieved less than 100 copies/ml concurrent with plasma viremia less than 10 000 copies/ml, followed by therapy discontinuation until CSF viral load reached greater than 1000 copies/ml, and then ART reinitiation. This was repeated for a total of one to three rounds of ART before a final 2–4-week period of ART was implemented in all animals prior to necropsy. Initiation of ART at week 3 and the short-term reinitiation before necropsy effectively decreased CSF SIV RNA. However, cell-associated viral RNA remained detectable in some CNS regions at necropsy, and viral DNA persisted throughout the CNS. Lastly, CNS CD4^+^ T cells were not fully restored in treated animals. Despite early treatment initiation, these data highlight virus persistence and poor CD4^+^ T-cell recovery in treated chronic infection. Although the suboptimal ART treatment plan should be considered when interpreting these data, the observations are consistent with the seeding of a persistent CNS viral reservoir.

A separate study focused on the impact of acute SIVmac251 infection on CNS myeloid cells [52^▪^], which are considered critical cellular harborers of HIV-1 DNA in the brains of PLWH [53]. Brain tissue from SIVmac251-infected macaques was isolated 12 days postinfection, when viral replication is active within the CNS, and 0.15% of myeloid and 1.4% of lymphocytes had detectable SIV transcripts. Single-cell RNA sequencing identified microglia and CNS-associated macrophage (CAM) clusters. Acute SIV infection altered the frequency of brain myeloid cells, with reduced levels of homeostatic and preactivated microglia clusters and elevated activated clusters relative to uninfected animals. Microglia from acutely infected animals also upregulated genes linked to immune-mediated defense and senescence. A less inflammatory CD16^hi^CD14^lo^ CAM cluster predominated in uninfected animals, while a more inflammatory CD16^lo^CD14^hi^ cluster predominated during acute infection. A subset of the elevated microglia and CAM clusters exhibited increased expression of genes linked to neurocognitive impairments. Finally, investigation of apoptosis-related genes revealed increased expression of the antiapoptotic CD5L marker in one of the microglia clusters expanded during acute SIV infection. These data highlight that acute SIV infection dramatically impacts CNS myeloid cell populations, and these alterations have the potential to contribute to immune activation, neurological disorders, and the persistence of an apoptosis-resistant reservoir in chronic infection.

To address the scarcity of therapeutics capable of crossing the BBB and targeting virus-infected CNS cells, Bohannon *et al.*[54^▪^] built on their previous observation that colony-stimulating factor 1 receptor (CSF1R) expression and signaling is increased in infected brain macrophages during SIVmac251 infection [55]. They leveraged a CNS penetrating CSF1R kinase inhibitor, BLZ945, to promote apoptosis of perivascular macrophages (PVMs) during acute SIV infection. SIVmac251-infected macaques were treated with a CD8-depleting antibody during acute infection to drive viral replication and cellular infection in the CNS, and were dosed with daily low (10 mg/kg) or high doses (30 mg/kg) of BLZ945 starting at day 10 postinfection and spanning 20–30 days. Treatment with the high dose reduced PVMs across each analyzed brain region compared to untreated controls. Additionally, BLZ945-treatment (i.e. high and/or low-dose) significantly decreased viral DNA-carrying cells in 9/11 brain regions assessed. This study demonstrates that therapeutics focused on accessing CNS targets can reduce the frequency of virus-infected cells.

The reviewed studies highlight the infection of CD4^+^ T cells and myeloid cells during acute SIV infection, the impact of acute infection on the frequencies and phenotypes of these cells, and the possibility of targeting infected cells in the CNS with drugs that penetrate the BBB. Additional research is needed to clarify the relative roles of CD4^+^ T cells and myeloid cells for importing viruses into the CNS and to identify BBB-penetrating drugs that can clear multiple infected cell types.

## STUDYING THE IMPACTS OF ACUTE AND CHRONIC INFECTION ON THE LIVER

As with neurocognitive impairment, ART-treated PLWH experience significant morbidity and mortality from liver disease [56]. The events contributing to liver disease in PLWH are poorly understood due to difficulties associated with collecting liver biopsies, as well as problems discerning the relative contributions of chronic ART use, co-infections, substance use, and viral and immune-mediated disease [57^▪^]. Until recently, NHP studies have not contributed to defining the impact of the distinct phases of SIV infection on the liver, as the tissue was only sampled during necropsies of infected animals.

Derby *et al.*[57^▪^] utilized a laparoscopic technique to sample the livers of SIVmac251-infected macaques longitudinally to determine how the liver is impacted throughout infection. Serial sampling preinfection and 2, 6, 16–20, and 32 (necropsy) weeks postinfection enabled detailed chronicling of liver pathogenesis. Infected animals exhibited high levels of viremia throughout the study. Viral DNA was also detected in the liver, achieving as much as 5 × 10^3^ copies per 10^6^ cells 2 weeks postinfection. Blood chemistry analysis revealed significantly higher levels of cholesterol and aspartate aminotransferase (AST) in infected animals compared to uninfected controls. These differences were most pronounced in acute infection. Histological analyses revealed increased microvesicular steatosis among acutely infected macaques, which resolved by 6 weeks postinfection. Assessments of liver tissue collected at necropsy identified sinusoidal dilatation, or widening of hepatic capillaries, plaguing most animals during chronic infection. Varying liver pathologies at different phases of SIV infection highlight the potential need to consider the timing of therapeutic interventions for protecting liver health in PLWH.

## CONTRIBUTION OF EVENTS DURING ACUTE INFECTION TO ACHIEVING VIRAL CONTROL

NHP models of HIV-1 infection are also valuable for assessing how the events of acute infection influence the natural control of viral replication or control following the interruption of ART. Among PLWH, those exhibiting these forms of viral control are known as elite controllers and posttreatment controllers (PTCs), respectively [58,59]. Class I MHC alleles are associated with spontaneous viral control [60,61]. In contrast, many PTCs lack class I MHC alleles linked to spontaneous viral control and even carry alleles related to the risk of disease progression (e.g. *HLA-B∗07* and *HLA-B∗35*) [62]. Early ART initiation that restricts viral reservoir size and preserves immune function appears to be important for viral control in many PTCs [58,63]. However, much remains unresolved about the factors contributing to natural and posttreatment viral control. Recent studies in NHP models illuminate aspects of early infection or interventions during this period that may contribute to achieving viral control.

Harwood *et al.*[64^▪▪^] developed an NHP model of PTCs using SIV-infected Mauritian cynomolgus macaques (MCMs). These animals exhibit limited genetic diversity and can carry MHC haplotypes associated with natural viral control [65,66]. However, additional mechanisms of viral suppression were explored by infecting animals lacking the protective M1 and M6 MHC haplotypes and carrying at least one copy of the M3 haplotype not associated with viral control [64^▪▪^]. ART was initiated 2 weeks after SIVmac239M infection and continued for 8 months. After treatment interruption, seven of eight animals exhibited viral control or transient viremia below 10^4^ copies/ml for 6 months. The authors noted that the high proportion of MCMs exhibiting posttreatment viral control is distinct from the almost universal viral rebound observed in SIV-infected rhesus macaques that initiate ART 1–4 weeks postinfection and undergo treatment interruption. Some differences in viral reservoirs and acute infection viremia were noted between these two species: MCMs harbored lower levels of intact proviral DNA, and while peak viremia is similar, it is more prolonged in rhesus. Early ART initiation during acute infection is likely significant for posttreatment viral control in MCMs, as four of six animals from a second cohort, including animals carrying the protective M1 haplotype that did not initiate ART until 8 weeks postinfection exhibited sustained viremia within 4 weeks of treatment interruption after 20 months of therapy.

Several lines of evidence indicated immune-mediated viral control after treatment interruption in the cohort that initiated ART 2 weeks postinfection. First, a rechallenge with an isogenic virus 25 weeks after ART interruption resulted in four animals rebounding or exhibiting transient viremia and four remaining aviremic. The aviremic animals had higher antiviral T-cell responses, as assessed by ELISPOT. Second, CD8α depletion 8 weeks after the viral rechallenge resulted in a 3–6 log_10_ viral rebound in all animals. Finally, when assessing MCMs from both cohorts that rebounded, blipped, or maintained viral control, the authors noted that lower frequencies of T cells expressing exhaustion markers was associated with posttreatment control. Given that PTCs are rare within the community of PLWH [58,63], an NHP model of this phenomenon represents a significant advance for studying correlates of sustained viral control in the absence of ART.

In a similar study, Passaes *et al.*[67^▪▪^] demonstrated that MCMs infected with SIVmac251 and treated with ART at 4 weeks postinfection were more likely than animals treated 24 weeks postinfection to achieve posttreatment control (82 vs. 18%) after 2 years of ART and 24–48 weeks of posttreatment follow-up. Animals were characterized as PTCs if they did not rebound, or if they achieved viremia less than 400 copies/ml at least one time after rebounding. Posttreatment control was linked to CD8^+^ T-cell viral suppressive activity during treatment interruption. The authors conclude that early ART allows the priming of a potent CD8 T-cell population that can survive throughout the treatment period and exhibit high proliferative capability during viral reactivation.

Additional efforts to probe mechanisms driving viral control in elite controllers studied 32 macaques carrying the protective *Mamu-B∗08* class I MHC allele [68^▪^]. Sixteen animals were immunized to induce CD8^+^ T-cell responses directed to SIVmac239 Vif and Nef. The vaccine regimen showed no efficacy in preventing SIVmac239 infection. However, virus control (defined as ≤10 000 viral RNA copies/ml for at least four consecutive weeks) was more common among vaccinated (13/16) than unvaccinated (7/16) animals. Viral load setpoint was not linked to the quantity or quality of vaccine-induced antiviral T-cell responses, measured via tetramer and intracellular cytokine staining, respectively. To assess the potential role of innate immune mechanisms in achieving elite control, the authors performed whole blood transcriptomics on the 32 *Mamu-B∗08*^+^ animals from the vaccine study and eight SIVmac239-infected *Mamu-B∗08*^*-*^ animals with progressive infections using specimens collected during acute infection. Viral control in *Mamu-B∗08*^*+*^ animals was associated with a rapid upregulation and resolution of gene expression related to innate immune responses.

The data from these studies highlight the importance of acute and early infection events for establishing viral control. Critical events include the timing of initiation of exogenous therapies (i.e. ART) and the development of endogenous immune responses. Continued research is required to determine if NHP models of spontaneous and posttreatment control can be extrapolated to PLWH, including developing optimized strategies to promote these phenotypes more uniformly in PLWH.

## THERAPEUTIC ANTIBODY INTERVENTIONS DURING ACUTE INFECTION

As with early initiation of ART during acute SIV infection, therapeutic antibody interventions during acute SHIV infection can alter the disease course [69–73]. Nishimura *et al.*[72] reported that a subset of macaques treated with two bNAbs – 10-1074 and 3BNC117 – during early acute SHIV-AD8-EO infection (starting at day 3) achieved long-term viral control. CD8^+^ T-cell responses mediated this control, as animals exhibited viral rebound following CD8^+^ T-cell depletion. The administered bNAbs were hypothesized to form immune complexes that initiated a vaccinal effect that established the antiviral CD8^+^ T cells. Vaccinal effects induced by immune complexes would involve antigen uptake and processing by antigen-presenting cells through the recognition of antibody Fc portions via Fc receptors.

Dias *et al.*[74^▪▪^] assessed the immunological and virological impacts of administering bNAbs mutated to enhance Fc receptor binding during acute infection. Macaques infected with SHIV-AD8-EO received infusions during acute infection consisting of either wildtype (WT) PGT121 and VRC07-523-LS bNAbs or versions of these antibodies mutated to enhance Fc receptor binding. Unlike Nishimura *et al.*, bNAb-treated animals did not exhibit prolonged viral control. However, treatment with bNAbs delayed the onset of viremia until 6 or 10 weeks postchallenge for enhanced Fc receptor binding or WT bNAbs, respectively, compared to 2 weeks in controls. The timing of the onset of viremia in the bNAb-treated animals corresponded with the disappearance of the bNAbs from plasma, which followed the development of antidrug antibodies. Early bNAb treatment also delayed the onset of detectable viral RNA in lymph nodes.

To assess the immunomodulatory effects of the bNAbs, Gag-specific CD8^+^ T cells in the periphery and lymph nodes were evaluated, along with gene set enrichment analysis on lymph node cells [74^▪▪^]. bNAbs with enhanced Fc receptor binding impacted several aspects of adaptive and innate cellular immunity, resulting in: increased frequency of CD69^+^IFNγ^+^ Gag-specific CD8^+^ T cells in peripheral blood at week 20 postchallenge; elevated pro-inflammatory signaling among lymph node monocytes and natural killer cells at week 8 postchallenge; and diminished TNF signaling through NF-κB in lymph node T cells at week 8 postchallenge.

Given their ability to modulate adaptive and innate cellular immunity, bNAbs with enhanced Fc receptor binding warrant further exploration. The potential of these products during acute infection is of particular interest, given the possibility of altering/attenuating chronic infection outcomes.

## CONCLUSION

**FIGURE 2:**
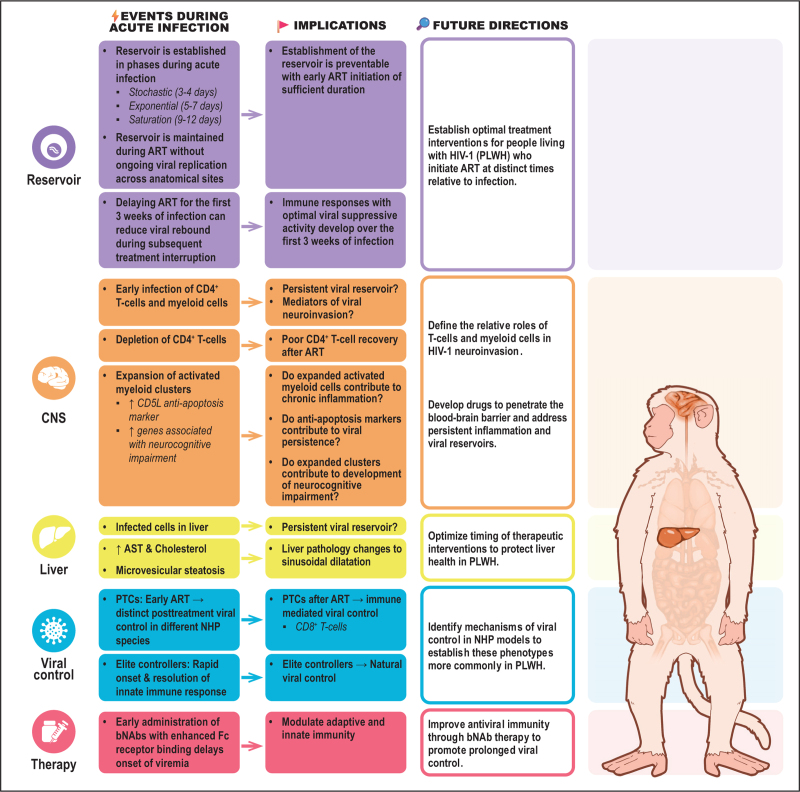
Summary of significant observations during acute infection in nonhuman primate models of HIV-1 infection reported in the manuscripts surveyed during the 18-month review period. The observations are linked with implications and directions for further study.

NHP models of HIV-1 infection continue to be valuable assets for complementing the study of AHI in PLWH. These models provide a parallel experimental pathway to investigate many aspects of AHI that cannot be addressed in clinical cohorts. As exhibited in Fig. 2, the highlighted studies from the review period represent experiments in NHP models demonstrating how acute infection events can impact short-term and long-term disease manifestations. Continued research in NHP models of HIV-1 infection will more precisely define the acute phase of the disease and generate hypothesis-driven strategies for therapeutic interventions aiming to cure or control chronic viral infection in the absence of ART.

## Acknowledgements

*We acknowledge Jason Thean Kit Ooi from the Henry M. Jackson Foundation for the Advancement of Military Medicine, Inc. (HJF) Global Infectious Diseases program for figure production. We thank Dr Sandhya Vasan (HJF and U.S. Military HIV Research Program) for critical review of the manuscript.*

*Disclaimers: Material has been reviewed by the Walter Reed Army Institute of Research. There is no objection to its presentation and/or publication. The opinions or assertions contained herein are the private views of the authors, and are not to be construed as official, or as reflecting true views of the Department of the Army or the Department of Defense.*

*The view(s) expressed herein are those of the author(s) and do not reflect the official policy or position of the Henry M. Jackson Foundation for the Advancement of Military Medicine, Inc.*

### Financial support and sponsorship

*M.S.P. is supported by a K01 award (K01OD031900) from the National Institutes of Health's Office of the Director.*

*This work was supported by agreements #W81XWH-18-2-0040 and #HT9425-24-3-0004 between the Henry M. Jackson Foundation for the Advancement of Military Medicine, Inc., and the U.S. Department of Defense (DOD).*

### Conflicts of interest

*There are no conflicts of interest.*

## REFERENCES AND RECOMMENDED READING

Papers of particular interest, published within the annual period of review, have been highlighted as:

▪ of special interest
▪▪ of outstanding interest
